# *Drosophila* symbionts in infection: when a friend becomes an enemy

**DOI:** 10.1128/iai.00511-24

**Published:** 2025-04-02

**Authors:** Yi Yu, Igor Iatsenko

**Affiliations:** 1 Research Group Genetics of Host-Microbe Interactions, Max Planck Institute for Infection Biology, Berlin, Germany; University of California at Santa Cruz Department of Microbiology and Environmental Toxicology, Santa Cruz, California, USA

**Keywords:** gut microbiota, infectious disease, endosymbionts, host-microbe interactions, *Drosophila*

## Abstract

The insect microbiome is comprised of extracellular microbial communities that colonize the host surfaces and endosymbionts that reside inside host cells and tissues. Both of these communities participate in essential aspects of host biology, including the immune response and interactions with pathogens. In recent years, our knowledge about the role of the insect microbiome in infection has increased tremendously. While many studies have highlighted the microbiome’s protective effect against various natural enemies of insects, unexpected discoveries have shown that some members of the microbiota can facilitate pathogenic infections. Here, we summarize studies in the fruit fly, *Drosophila melanogaster*, that have substantially progressed our understanding of host-pathogen-microbiome interactions during infection. We summarize studies on the protective mechanisms of *Drosophila* gut microbiota, highlight examples of microbiome exploitation by pathogens, and detail the mechanisms of endosymbiont-mediated host protection. In addition, we delve into a previously neglected topic in *Drosophila* microbiome research—the crosstalk between endosymbionts and gut microbiota. Finally, we address how endosymbionts and gut microbiota remain resilient to host immune responses and stably colonize the host during infection. By examining how the microbiome is influenced by and reciprocally affects infection outcomes, this review provides timely and cohesive coverage of the roles of *Drosophila* endosymbionts and gut microbiota during infections.

## INTRODUCTION

Occupying the interface between host and environment, host-associated microbes play essential roles in interactions with pathogens and influence disease progression (1–4). Considering that most entry sites for pathogens into the host organism are colonized with microbiota (5), pathogen-commensal interactions are an inevitable and fundamental aspect of the disease.

The importance of such interactions is exemplified by colonization resistance—a concept of protection of the host from pathogens by commensal microbes (6–9). Colonization resistance is a widely observed phenomenon in many organisms and can be direct or indirect. Direct colonization resistance occurs when intestinal microbiota directly suppresses the pathogen via nutrient competition or secretion of antimicrobial molecules, like bacteriocins or organic acids (10–16). Indirect colonization resistance occurs when commensals protect the host by modulating the intestinal immune responses to increase the resistance to infection. One mechanism of modulation is the induction of the expression of intestinal anti-microbial C-type lectins (17–20). The protective role of commensals is well studied, and a plethora of underlying mechanisms have been identified. However, studies of different systems have implied that microbiota may take on a pathogenic rather than protective role, where they promote intestinal infections (1, 21–23).

Commensals and their derived signals and nutrients can be hijacked by various enteric pathogens to promote disease and coordinate the expression of the virulence repertoire (1, 4).

The metabolic interplay between microbiota and bacterial pathogens is frequently identified as an underlying cause of altered virulence of a bacterial community. For example, *Salmonella enteric*a ser. Typhimurium feeds on microbiota-derived hydrogen to invade the gut ecosystem (24). Pathogens can also make use of host-derived metabolites liberated by commensals to modulate pathogenesis. For instance, *Bacteroides thetaiotaomicron* releases fucose and sialic acid from host glycans and produces high levels of succinate, which are necessary for pathogen expansion in the mammalian intestine and are sensed by enterohemorrhagic *Escherichia coli (EHEC), S*. Typhimurium*,* and *Clostridium* (25–27). Moreover, a recent study showed that *B. thetaiotaomicron*, through the digestion of dietary pectin, releases galacturonic acid which is used by EHEC and *Citrobacter rodentium* in the gut as a carbon source, aiding pathogens’ initial expansion (28). Beyond providing nutrients to pathogens, commensal-derived metabolites can signal the regulation of virulence factor production and ultimately affect the progression of disease. For example, microbiota-produced ethanolamine is used as a nitrogen source and a regulator of virulence genes by EHEC, *S*. Typhimurium, and *Listeria monocytogenes* (29–32). Similar effects were reported for a number of other metabolites, like succinate, acetate, butyrate, and taurocholate (33–38).

Beyond these few exceptions, we are still largely in the dark when it comes to the molecular mechanisms of how commensals facilitate infections.

Given the complexity of animal microbiota, one of the main challenges is to identify the microbiota member or community implicated in positive or negative interactions with a particular pathogen. Since the vast majority of mammalian microbiota remains uncultivable and unamenable to genetic manipulation, functional validation of suspected interactions is not experimentally feasible, and many studies remain correlative.

The fruit fly, *Drosophila melanogaster*, with its extensive genetic toolkit, evolutionary-conserved innate immune defense, and genetically tractable microbiome, represents an ideal model to address an ambitious question of the mechanistic role of commensals in facilitating intestinal infections (39–44).

Several distinct attributes of *Drosophila* microbiota stand behind the successful use of the fruit fly model in microbiome research. Genetic tractability and cultivability of the *Drosophila* microbiota members combined with the simple taxonomic composition allow functional studies of the molecular mechanisms of commensal-host interactions (41, 45–47). The investigation into the host component of these interactions is facilitated by a wealth of genetic, genomic, and molecular resources available in *Drosophila* (48, 49). Another particular advantage of the fruit fly model is the simplicity of generating and maintaining germ-free, or axenic, animals. Moreover, gnotobiotic animals colonized with a defined microbiota can be generated easily (45, 49).

Due to these exceptional features, *Drosophila* models have been widely utilized to investigate the effect of host-associated microbes on host physiology, including interactions with pathogens (23, 47, 49–54).

This review aims to provide timely and unified coverage of the role of *Drosophila* gut microbiota and endosymbionts in infection.

## COMPOSITION AND ESTABLISHMENT OF *DROSOPHILA* MICROBIOTA

In laboratory and field settings, *Drosophila melanogaster* is colonized by relatively simple microbial communities comprising 2–30 species, belonging to the Proteobacteria and Firmicutes phyla. These communities are represented by two dominant families *Acetobacteraceae* and *Lactobacillaceae*, and by minor families *Enterococceae* and *Enterobacteriaceae* (55–60). The following species are the most consistently associated with flies across studies: *Lactiplantibacillus plantarum*, *Levilactobacillus brevis*, *Acetobacter pomorum*, *A. pasteurianus*, and *Enterococcus faecalis* (42, 46, 47, 49, 61, 62). This community, rich in lactic acid and acetic acid bacteria, reflects the fermentative substrates consumed by flies (42, 63). The *Drosophila* microbiota composition is significantly affected by the fly diet; the continuous ingestion of microbes from the food is crucial for the establishment and maintenance of intestinal commensals in *Drosophila*. The majority of fruit fly intestinal commensals cannot stably colonize the gut and must be regularly reintroduced through re-ingested food (61, 64, 65).

Newly emerged flies initially harbor a low number of microbes in their gut. However, within the first day of their adult life, these flies acquire microbiota by consuming bacteria from food contaminated with their parents’ feces (61, 64). Moreover, female flies pass on their microbiota to their offspring by depositing microbes on the eggshells. Upon hatching, larvae become colonized by ingesting the bacteria-rich eggshell and feeding on the microbe-laden food (62, 65). These interactions among *D. melanogaster*, microbiota, and nutrition likely contribute to the significant variability in microbiota composition and density observed among individual flies raised in the same culture vial (66, 67). The bacterial load can differ by up to one logarithmic unit between flies cohabiting in the same environment (61). Furthermore, flies that are frequently transferred to sterile food, preventing re-ingestion of microbes with the diet, can lose their microbiota and become germ-free (64, 68). Further importance of the food substrate in *Drosophila*-microbiota interactions is illustrated by the finding that the diet, rather than the host, is the major force driving the evolution of symbiotic properties of the prominent fly commensal *L. plantarum* (69).

While the transitory association between *Drosophila* and its commensals holds true for bacterial isolates from *Drosophila* laboratory stocks, some of the bacteria isolated from wild-caught *D. melanogaster* can stably persist and proliferate in the gut. Such stable association confers fitness advantages for both partners in an ecological context (68). Recent studies started to address the mechanisms of microbial stable colonization using *L. plantarum* isolated from a wild-caught fly, which persists in the *Drosophila* foregut. Dodge et al. discovered a precise, spatially defined, physical niche within the adult *Drosophila* foregut, including the proventriculus, the crop, and the crop duct that is specifically colonized by wild strains of *Lactobacillus* (70). Subsequent work demonstrated that *L. plantarum* colonizes its niche through host-specific serine-rich repeat protein adhesins encoded by genes carried on a colonization island (71, 72). Beyond uncovering the basis of niche-specific colonization, this work together with the other studies highlights the importance of intra-strain variation in conferring the host phenotypes (67, 68, 73, 74). Differences among microbial strains are often neglected and could explain some of the conflicting results in the field (75).

## BENEFICIAL AND DETRIMENTAL ROLE OF *DROSOPHILA* MICROBIOTA DURING INFECTIONS

*Drosophila* microbiota affects essentially every aspect of the host physiology, including development, behavior, lifespan, and disease resistance. While these topics were reviewed previously (46, 47, 54, 62, 76), here we would like to focus specifically on the microbiome’s role during infection—an emerging topic that received little attention in previous reviews.

**Fig 1:**
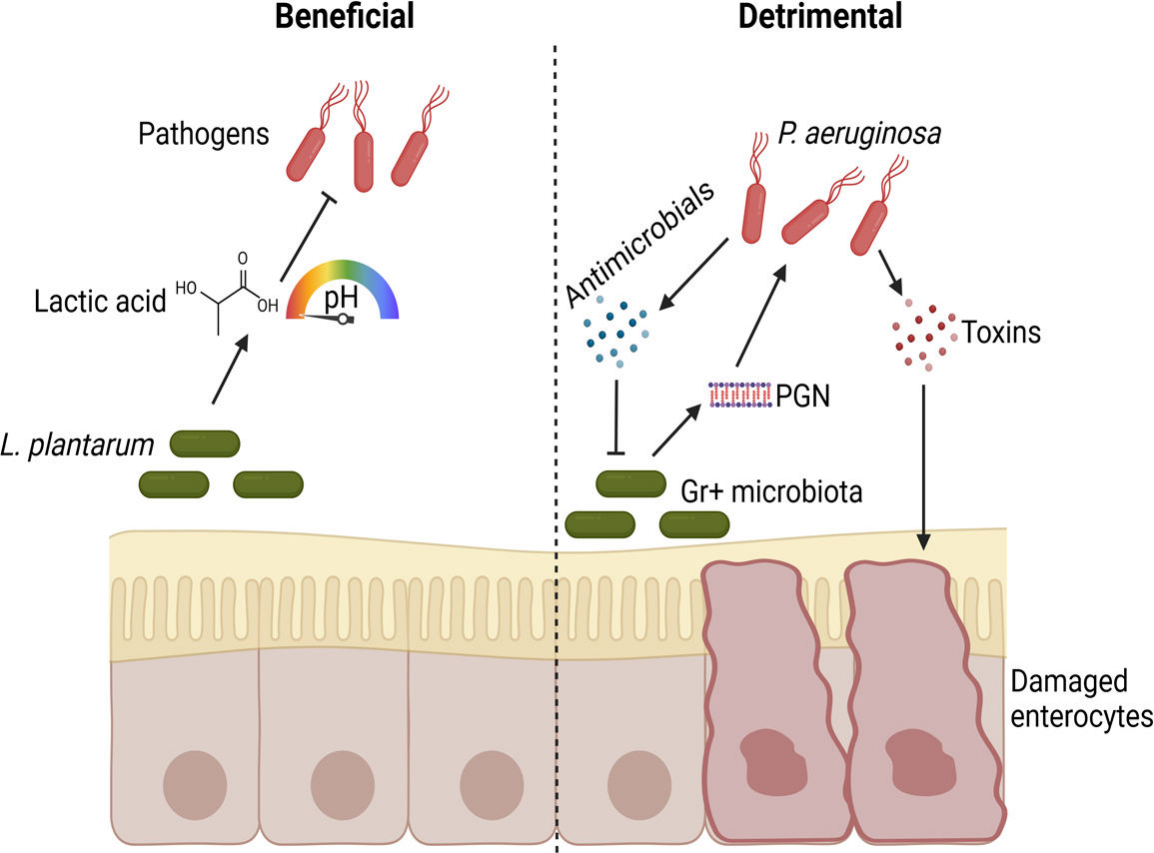
Examples of beneficial and detrimental effects of the microbiota during infection in *Drosophila*. Beneficial: *L. plantarum* produces lactic acid, which acidifies the environment and specific regions of the *Drosophila* gut, inhibiting pathogen growth. Detrimental: peptidoglycan (PGN) released by Gram-positive microbiota activates toxin and antimicrobial production in *P. aeruginosa*, leading to epithelial damage and suppression of the microbiota. Created in BioRender. Iatsenko, I. (2025) https://BioRender.com/b61j017

Consistent with the well-described protective role of host microbiota in different organisms against pathogens and parasites, some *Drosophila*-associated commensals exhibit a defensive role. For instance, Blum et al. (64) demonstrated that *L. plantarum* improved *Drosophila* survival in the presence of pathogens *Pseudomonas aeruginosa* or *Serratia marcescens,* but the mechanism of this protection has not been explored. A recent study proposed that gut microbiota may protect the host from invasive microbes through environmental acidification. Specifically, the production of lactic acid by *L. plantarum* through lactate dehydrogenase creates an acidic environment that inhibits the growth of invasive pathogens (77). Lactic acid produced by *L. plantarum* was also implicated in the inhibition of and fly protection against a newly described fungus: *Diaporthe* FY (78). *Acetobacter pomorum* might similarly protect flies via acetic acid-mediated inhibition of fungi (79). This mechanism could potentially explain the increased survival likelihood of microbiota-colonized as compared to axenic *Drosophila* larvae after *Candida albicans* infection (80). Thus, acid-mediated pathogen inhibition by the microbiota emerges as a gatekeeper against invading pathogens in *Drosophila* (81) (Fig. 1).

In addition to gut microbiota, *Drosophila* surface-associated microbes can protect the flies against fungal infections. While the *Drosophila* surface microbiota remains underexplored, several studies have demonstrated that fly external surfaces serve as battlegrounds for microbiota-pathogen competition. Hong et al. showed that surface bacteria could defend flies against fungal infections. *L. plantarum* specifically was shown to antagonize fungal spore germination and significantly delay fungal infection of axenic flies (82). Intriguingly, fungus that lacks defensin-like antimicrobial gene *BbAMP1* and is thus not able to inhibit insect surface microbiota was impaired in virulence in gnotobiotic but not in axenic flies (83). This finding suggests that the ability of fungi to compete with microbiota is essential for virulence. In line with this, the entomopathogenic fungus *Metarhizium robertsii,* engineered to express the antibacterial moricin gene, showed a substantially enhanced ability to kill insects. This effect was due to the ability of fungus to suppress insect cuticular bacteria and to disrupt the gut microbiome. Specifically, an overgrowth and translocation to the hemolymph of the opportunistic pathogens of *Providencia* species was detected and shown to contribute to insect death (84). A very similar scenario was described in mosquitoes: fungal infection with *Beauveria bassiana* caused dysbiosis of mosquito gut microbiota. In particular, overgrowth of the opportunistic pathogenic bacterium *Serratia marcescens* in the midgut and translocation to the hemocoel were identified as leading causes of mosquito death (85). These two examples with fungal infections illustrate that microbiota might be exploited by some pathogens rather than serve as a protective barrier. Cases of infection facilitation in flies by microbiota were also observed with bacterial pathogens. For example, *Pseudomonas aeruginosa* virulence was increased by the microbiota in flies, resulting in increased death of microbiota-colonized versus axenic flies. Mechanistically*, P. aeruginosa* senses peptidoglycans shed by gram-positive bacteria and responds to this cue through the enhanced production of virulence factors (86) (Fig. 1). The pathogenesis of *Vibrio cholerae* was also increased by microbiota in *Drosophila* models (52). Specifically, Fast et al. found that *Vibrio cholerae* T6SS contributes indirectly to *Drosophila* death. T6SS-dependent killing of the host required the presence of gut commensal *Acetobacter pasteurianus* (87). Subsequent work showed that *V. cholerae* causes damage to the midgut epithelium and prevents compensatory epithelial renewal. This destruction is dependent on microbiota, as elimination of the intestinal commensals restores epithelial renewal capacity in infected intestines (88). Collectively, these findings suggest that microbiota facilitate *V. cholerae* infection by enabling T6SS-dependent inhibition of epithelial renewal.

Given that *Drosophila* microbiota modulates several immune and repair signaling pathways in the gut, including the generation of reactive oxygen species (ROS), immune deficiency (IMD) pathway, Janus kinase/Signal transducers and activators of transcription (JAK/STAT) pathway, and c-Jun NH2-terminal kinase (JNK) pathway (43, 44, 76, 89), it is very likely that microbiota impact the outcome of infections by regulating these pathways. For instance, the altered susceptibility of axenic flies to infections could be due to dampened immune or repair pathway activation. Such an indirect role of microbiota in infection via alteration of the host is well established in other models but still needs to be investigated in *Drosophila*.

## THE ROLE OF DIET IN *DROSOPHILA* INTERACTIONS WITH MICROBIOTA AND PATHOGENS

Diet is a well-recognized factor affecting host physiology and host interactions with pathogens and microbiota (90, 91). The diet composition was shown to have a major impact on the structure and abundance of fly microbiome (92, 93). For instance, an increase in yeast concentration led to a substantial increase in the total abundance of gut microbes but decreased their alpha diversity (94). Another study associated a yeast-rich diet with a high abundance of *Enterobacteriaceae* (63). By contrast, studies that explored the effect of high-sugar diets on the microbiome reported an increase in microbiota diversity (95) and a high prevalence of *Providencia species* (63). Raising flies on a diet supplemented with casein shifted the microbiota composition to predominantly *Lactobacillus* species (95)

Several studies that investigated the effect of a high-fat diet on microbiota reported an overall increase in gut microbiota abundance when flies were fed fat-rich food (96–98). The dominant species, however, differ among studies. Wang et al. found that a high-fat diet significantly increased the abundance of *Acetobacter malorum* in the gut (96), while von Frieling detected significant enrichment of orders *Enterobacteriales* and *Caulobacterales* upon high-fat diet feeding (97).

Diet composition is also known to affect *Drosophila’s* susceptibility to infections (99). For example, flies fed high-sugar diets are more susceptible to infections by the Gram-negative pathogens *Providencia rettgeri* and *Serratia marcescens* (100, 101). Yeast-rich diets are often associated with the increased survival of insects after infection (102), while protein shortage has been reported to negatively affect survival after infections (103, 104). These dietary interventions were shown to affect the susceptibility to infections by altering the host defenses. The potential role of microbiota in mediating dietary effects on infection outcomes was mostly neglected. However, given that diet influences microbiota and host susceptibility to infections, diet, besides its direct impact on the host, might affect infection susceptibility indirectly by altering host microbiota. The link between diet-induced changes in fly microbiome and susceptibility to infections remains to be demonstrated. Furthermore, the impact of diet on microbiota complicates comparisons between different studies that utilized different media. For example, microbiota was reported to protect flies against (64) but also support the infection with the pathogen *P. aeruginosa* (86). Such discrepancies could be attributed to diet-driven variations in fly microbiota between labs. Therefore, the importance of diet in *Drosophila*-microbe interactions should not be underestimated, and precise descriptions of diet composition and microbial strains should always be reported.

## DEFENSIVE ENDOSYMBIONTS OF FRUIT FLIES

In addition to microbial communities colonizing the gut and external surfaces, insects frequently harbor endosymbionts: symbiotic microbes that live inside host cells or tissues. *Wolbachia* and *Spiroplasma* are the only known endosymbionts of *Drosophila* (59, 105, 106). *Wolbachia* are intracellular Alphaproteobacteria that are transmitted vertically through host eggs and cause reproductive manipulations (107, 108). *Spiroplasma* are gram-positive, helical bacteria devoid of cell walls, which belong to an ancient lineage of host-associated Mollicutes (105, 109, 110). *Spiroplasma* live extracellularly in the host hemolymph where they feed on host lipids (111, 112). One of the most prominent phenotypes conferred by *Wolbachia* and *Spiroplasma* is host protection against natural enemies (113–115). *w*Mel, the *Wolbachia* strain present in *Drosophila melanogaster*, provides strong protection against multiple RNA viruses, which could offer a fitness benefit in nature (116–118). *Wolbachia*’s antiviral properties are exploited in the control of dengue and Zika virus transmission by mosquito vectors (119). The release of *Wolbachia*-infected *Aedes aegypti* mosquitoes was shown to reduce the number of dengue cases in affected areas of the world (120, 121). Although the molecular mechanisms of *Wolbachia*-mediated antiviral protection remain unknown, the following mechanisms have been proposed: immune priming (122), increased ROS production (123), and competition for resources between symbiont and pathogen (124, 125). In addition, several *Wolbachia* strains with different degrees of protection have been isolated. Generally, strains that reached higher titers in the host conferred higher antiviral protection (126, 127), demonstrating a correlation between *Wolbachia* abundance and protection. However, further mechanistic studies of *Wolbachia*-mediated protection remain challenging as this endosymbiont cannot be cultured outside of host cells or genetically manipulated. Although *Wolbachia*-mediated antiviral protection attracted a lot of attention, the potential role of *Wolbachia* in interactions with other types of pathogens, including fungi and bacteria, has been little studied. A recent study demonstrated that *Wolbachia* can confer protection against several but not all tested fungal and yeast pathogens. Host sex, genetics, and pathogen species were identified as significant determinants of each infection outcome (128). Further research is required to understand the variable ability of *Wolbachia* to inhibit fungal pathogens and the mechanistic basis of antifungal protection. Regarding antibacterial protection, several studies reported no significant effect of *Wolbachia* on *Drosophila* survival or ability to control pathogen growth after systemic infections with different bacteria (129, 130). One study, however, demonstrated that *Wolbachia*-carrying flies exhibited reduced mortality after enteric but not systemic infection with *Pseudomonas aeruginosa* (131). Thus, the route of infection is an important determinant of *Wolbachia* antibacterial protection in *Drosophila*. It will be important to investigate how such protection is achieved and how common it is among different endosymbionts.

Another endosymbiont of *Drosophila, Spiroplasma poulsonii*, has attracted research interest for its ability to protect flies against parasites such as nematodes and wasps (132). The protective effect against nematodes was initially observed in *Drosophila neotestacea*, a species commonly infected by the generalist nematode *Howardula aoronymphium*. Flies carrying a specific strain of *S. poulsonii* exhibit resistance to nematode infections, quickly outcompeting their symbiont-free counterparts in natural populations throughout North America (133). Notably, only the indigenous *S. poulsonii* strain from *D. neotestacea* demonstrated efficacy in shielding flies from nematode invasion. Strains originating from other hosts failed to provide protection, indicating variations in the defensive capabilities of *S. poulsonii* strains.

Moreover, multiple strains of *S. poulsonii* derived from *D. hydei, D. melanogaster*, and *D. neotestacea* have been documented to protect against parasitoid wasps from diverse lineages (134, 135). The toxins generated by *S. poulsonii* play a pivotal role in host defense against both nematodes and wasps (132, 136–138). Specifically, *Spiroplasma* produces ribosome-inactivating proteins (RIP) that target nematode 28S rRNA, disrupting an essential adenine base in a crucial loop structure necessary for translation initiation. This action irreversibly hampers protein synthesis, instigates apoptosis, and ultimately leads to necrosis. *In vitro* experiments with purified *Spiroplasma* RIP toxin showed depurination of nematode rRNA, while nematodes within *Spiroplasma*-infected flies exhibited pronounced signs of RIP-induced rRNA modification (137, 139). Apart from toxin-mediated parasite eradication, the competition between *Spiroplasma* and wasps for host lipids has been identified as a significant fly defense mechanism (140). Recently, we expanded the known defensive spectrum of *Spiroplasma* beyond wasps and nematodes by revealing a previously unappreciated role of *Spiroplasma* in host protection against bacterial and fungal pathogens (141). We identified Transferrin-mediated iron sequestration (142) and enhanced melanization (143) induced by *Spiroplasma* as a key mechanism underlying protection. Beyond protection, we discovered that symbiont-harboring flies were more susceptible to systemic infection with a specific pathogen, *Pseudomonas entomophila* (141). A negative effect of *Spiroplasma* on fly survival was also reported for infections with *E. carotovora* and *Enterobacter cloacae* (112). These cases illustrate that symbionts, similar to some gut commensals, might facilitate certain infections. Thus, specific pathogens might benefit from the symbiont-induced alterations in host physiology. Given that flies can simultaneously harbor both *Spiroplasma* and *Wolbachia*, it will be important to investigate how the two endosymbionts together affect the host’s ability to fight infections. One study that attempted to address this question reported that flies carrying both *Wolbachia* and *Spiroplasma*, and those containing single symbionts only had similar survival rates after infection with *P. luminescens* or *Escherichia coli* bacteria (144). Future studies should expand the spectrum of tested pathogens and include cases where the protective effect of either symbiont is known. For example, whether the antiviral effect of *Wolbachia* will be affected by *Spiroplasma* should be investigated. Considering recent advances in *Spiroplasma* transformation and *in vitro* culture (145, 146), with further methodology developments, this endosymbiont holds great promise of becoming fully genetically tractable. Combined with the power of fruit fly genetics, a *Drosophila-Spiroplasma* model might offer unique genetic manipulation opportunities and the ability to study both partners involved in endosymbiosis.

## CROSSTALK BETWEEN ENDOSYMBIONTS AND GUT MICROBIOTA

The *Drosophila* holobiont contains two groups of microbes: endosymbionts (*Wolbachia* and *Spiroplasma*) residing in host cells and tissues, and extracellular host-associated microbes colonizing the gut and other host surfaces. While endosymbionts and extracellular microbes collectively form the host’s microbiota, they are often treated and studied as distinct entities. Historically, the research on host-microbiota interactions has been focused on binary interactions either between host and symbiont or between host and microbiota. While such a reductionist focus provided important insights into the role of symbionts and microbiota in host physiology, it neglected interactions between endosymbionts and microbiota and how these interactions impact the host and interacting microbes (147). A holistic approach that covers the interactions between symbionts, host, and the remaining microbiota is challenging as it requires a model where the elimination of endosymbionts is possible without affecting the microbiota and vice versa. The fruit fly is one such model that has been used to study the interactions between endosymbionts and gut microbiota.

Several studies investigated the impact of *Wolbachia* on gut microbiota in *Drosophila* and other insects. In most cases, the presence of *Wolbachia* was correlated with reduced taxonomic diversity of microbiota and changes in the abundance of certain microbiota members. For example, Ye et al. reported that the presence of *Wolbachia* not only reduced microbiome diversity in the fly gut without affecting the total bacterial quantity but also led to the increase in abundance of *Leuconostocaceae* and *Acetobacteraceae* families (148). Recent work similarly reported that *Wolbachia* promotes extracellular microbiome growth. Specifically, colony-forming units of *Acetobacter* and *Lactobacillus* species in the presence of *Wolbachia* were 7.07-fold and 9.78-fold higher compared to symbiont-free flies (149). Another study, however, reported the opposite effect and found that *Wolbachia* bacteria reduce the proportion of *Acetobacteraceae* and specifically *A. pasteurianus* levels in gnotobiotic organisms (150). The abundance of Proteobacteria, especially *Acetobacter*, was reduced in the *Wolbachia*-infected *Drosophila nigrosparsa,* while *those of* Bacteroidetes and Actinobacteria were significantly increased (151). While the reduced diversity of resident bacteria in the presence of *Wolbachia* is consistent across studies and insects, including *Nilaparvata lugens* (152), *Aedes aegypti* (153, 154), *Sogatella furcifera* (155), and D. melanogaster (156), abundance even of the same microbiota species is differently affected across studies. Such discrepancies could be due to variables that differed among studies, including environment, host, and symbiont genetics. Thus, more controlled investigations are needed to conclusively establish the relationships between *Wolbachia* and intestinal microbial communities as well as the mechanisms that regulate them.

A few publications also aimed to address a reciprocal question: what is the effect of microbiota on *Wolbachia* abundance? Again, there was no clear consensus as the presence of gut microbiota was shown to both increase (149) and decrease (148) *Wolbachia* densities.

In addition, relationships between the two *Drosophila* endosymbionts, *Wolbachia* and *Spiroplasma* have been explored. In *D. melanogaster*, coinfection with the endosymbiont bacterium *Spiroplasma* reduced *Wolbachia* density, while *Spiroplasma* numbers remained unaffected by the presence of *Wolbachia* (157). In a different *Drosophila* species, *D. neotestacea, Wolbachia* abundance did not differ significantly between flies that bore or lacked *Spiroplasma. Spiroplasma* quantity, however, was increased in the presence of *Wolbachia,* indicating that *Wolbachia* promotes *Spiroplasma* populations. However, this effect is not reciprocated by *Spiroplasma* (158).

The increasing appreciation of symbiont-microbiota crosstalk in host physiology is evident in recent publications. Nevertheless, this field remains underexplored, with our understanding of the intricate interactions still limited. Many unanswered questions underscore the need for further exploration. Could crosstalk between endosymbionts or endosymbionts and microbiota explain some of the phenotypes conferred by microbiota or endosymbionts? Can symbionts affect the host physiology by modulating microbiota and vice versa? For example, can the protective effect of *Wolbachia* specifically against oral but not systemic *Pseudomonas* infection (131) be mediated by the *Wolbachia*-triggered changes in gut microbiota? Can symbionts and microbiota synergize in certain effects, like in host protection, by providing different mechanisms of protection? These are some of the exciting questions that await further investigation.

## HOST-SYMBIONT HOMEOSTASIS DURING INFECTION

The host-associated microbial communities are frequently exposed to defense responses induced by pathogens. Immune defense mechanisms are often non-specific and target conserved molecular patterns present in both pathogens and symbionts, raising the question of how symbionts endure such immune responses and stably colonize the host (159, 160). In the case of many endosymbionts, spatial separation of symbionts and immune responses could explain this phenomenon. Endosymbionts live inside host cells, tissues, or specialized symbiotic organs called bacteriomes which protection the host immune effectors (161, 162). How extracellular endosymbionts, like *S. poulsonii*, that reside in the host hemolymph endure the action of host immune effectors remains to be investigated. Intestinal microbial communities similarly reside in an open ecosystem offering no physical barriers against the host immune molecules. Although the *Drosophila* gut is compartmentalized into regions, some of which are not immune responsive (163), we found that gut bacteria localize in regions with strong immune activity (164), suggesting that they do not simply avoid immune defenses. Consistent with our findings, the symbiotic niche in the *Drosophila* gut—the proventriculus—is a gut region preferentially colonized by microbiota (70) despite undergoing a strong immune response (163, 165). Thus, gut symbionts colonizing the niche should have mechanisms to withstand the host’s immune defenses. In our recent papers, we provided the first insights into these mechanisms. Our first finding was that *Drosophila* microbiota composition and abundance were not significantly affected by the host immune responses triggered by intestinal infection. One microbiome member, *L. plantarum*, even increased in abundance after infection (164). We used *L. plantarum* as a model to investigate the mechanisms of commensal resilience in an inflamed gut environment. Given that antimicrobial peptides (AMPs) are the major immune effectors in *Drosophila* (40), we pursued a hypothesis that intrinsic resistance to AMPs allows *L. plantarum* to stay in the gut during infection. Consistent with this hypothesis, *in vitro* experiments confirmed *L. plantarum* resistance to several AMPs and antibiotics resembling AMP action. In a genetic screen, we identified several *L. plantarum* mutants sensitive to AMPs. The identified mutants were impaired in different processes like peptidoglycan O-acetylation, teichoic acid D-alanylation, or synthesis of lysyl-phosphatidylglycerol (164, 166). All of these disruptions led to increased negative cell surface charge and higher affinity to cationic AMPs. Our subsequent *in vivo* experiments demonstrated that in wild-type flies, AMP-sensitive mutants were eliminated from the gut following infection. However, in AMP-deficient flies, these mutants persisted, indicating that the ability to resist host AMPs is crucial for the resilience of commensals in an infected gut environment (164, 166). Given a similar finding in human commensal *Bacteroidetes* (167), resistance to host AMPs might be a common mechanism of microbiota persistence during infection. These results, together with the fact that resistance to host AMPs is one of the major virulence factors of several pathogens (168, 169), suggest that host-symbiont and host-pathogen interactions are mediated by the same molecular principles (170, 171).

We found that some *Drosophila* gut commensals, like *Acetobacter sp.*, are susceptible to AMPs *in vitro*. These findings agree with *in vivo* results demonstrating that the abundance of *Acetobacter sp.* is increased in ∆*AMP* mutant flies, supporting the crucial role of AMPs in controlling *Acetobacter* species (172). Although AMPs play a significant role in shaping the *Drosophila* microbiota, it has been demonstrated that the microbiota itself is the primary factor driving the evolution of *Drosophila* AMPs (173, 174).

In addition, non-inherited mechanisms might contribute to microbiota resilience. For example, microbiota exhibit rapid transcriptional reprogramming in response to host immune activation. Given that such transcriptional response includes upregulation of stress response-related genes, adaptation on a transcriptional level might be part of the microbiota resilience program (175). Furthermore, considering that *Drosophila* commensals under homeostatic conditions induce mild AMP response as compared to the pathogens (163, 165), exposure to such sub-lethal concentrations of AMPs might prime microbiota and increase tolerance to the high AMP concentration produced during infection (176). Exposure to low pH in the acidic region of the gut might also prime microbiota and increase the resistance to AMPs, as was demonstrated in *Vibrio fischeri* (177).

Besides AMPs, fruit flies produce additional immune effectors during infection. Specifically, reactive oxygen species (ROS) (178, 179) and iron-sequestration molecules (142) produced target commensals as well as pathogens. While the effect of ROS and iron limitation on microbiota was investigated in different systems (180–182), it remains to be studied how *Drosophila* microbiota is affected by and withstands these defense reactions.

## FUTURE DIRECTIONS

Despite recent advances in our understanding of the role of endosymbionts and microbiota in *Drosophila* interactions with pathogens, there are still many outstanding questions that remain to be addressed. Specifically, in many cases, the mechanisms of *Wolbachia*-conferred host protection remain unknown due to genetic intractability and uncultivability of *Wolbachia*. Although *Wolbachia* can be propagated in insect cell lines (183), genetic manipulation in cell culture is technically challenging, necessitating the development of axenic (cell-free) culture. In contrast to other endosymbionts, like *Spiroplasma*, that were axenically cultured, *Wolbachia* has a strongly degenerated genome and lacks almost all biosynthetic pathways to produce amino acids *de novo* and has retained only incomplete pathways for the synthesis of vitamins and cofactors (108, 184). Consistent with intracellular lifestyle and strong dependence on the host, *Wolbachia* requires fastidious growth conditions in terms of nutritional requirements and physicochemical environment (temperature, pH, and oxygen levels). Optimization of these culture conditions in combination with novel culture techniques (e.g., microfluidics) is necessary to advance mechanistic studies of *Wolbachia*-host interactions.

Another aspect that deserves particular attention is the need to move beyond the bacteria-centric view of *Drosophila* microbiota and expand our studies to non-bacterial components of the fruit fly microbiota. For example, various yeast species are commonly isolated from the *Drosophila* gut and food substrates and can affect the host in multiple ways, ranging from nutrient provisioning to behavior modulation (63, 185, 186). Similar important functions were reported for the fungal microbiota of fruit flies (187). The role of these fungal and yeast communities during infections has not been investigated yet. Furthermore, the most abundant inhabitants of the animal gut, bacteriophages, remain uncharacterized in *Drosophila*. Besides a single metagenome-based study (188) reporting the presence of sequences from potentially novel bacteriophages that could target major gut bacteria of *D. melanogaster—*including *Lactobacillus*, *Acetobacter*, and *Gluconobacter—*the phages of the *Drosophila* gut microbiota await their discovery. An enticing hypothesis that remains untested is whether phages could affect *Drosophila* physiology, including infection outcome, by modulating the microbiota. Another important aspect that needs to be considered is the role of interspecies interactions within the microbiota of the host. Studies that investigated pathogen-microbiota interactions in *Drosophila* primarily used gnotobiotic animals colonized with a single specific gut microbe. While successful in many cases, such a reductionist approach likely overlooked the contribution of interactions between microbiota members to infection outcomes (79, 189). Finally, the observations that male and female flies exhibit differences in microbiota composition (190, 191), that the microbiota influences infection outcomes, and that there is sexual dimorphism in infection susceptibility (192) raise an intriguing possibility that sex differences in microbiota communities contribute to sex dimorphism in infection susceptibility. Overall, the *Drosophila* model of pathogen-microbiome interactions offers many exciting avenues for future investigations.
